# Surgical treatment of breast cancer in patients aged 80 years or older – how much is enough?

**DOI:** 10.1186/1471-2407-14-700

**Published:** 2014-09-23

**Authors:** Nikola Besic, Hana Besic, Barbara Peric, Gasper Pilko, Rok Petric, Jan Zmuc, Radan Dzodic, Andraz Perhavec

**Affiliations:** Department of Surgical Oncology, Institute of Oncology, Zaloska 2, SI-1000 Ljubljana, Slovenia; Department of Surgical Oncology, Institute of Oncology and Radiology of Serbia, Pasterova 14, 11000 Belgrade, Serbia

**Keywords:** Breast cancer, Elderly, Treatment, Prognosis

## Abstract

**Background:**

The population of elderly people is increasing and so is the population of breast cancer patients aged ≥80 years. The aim of our retrospective study was to identify independent prognostic factors for the duration of breast cancer-specific survival of surgically treated patients aged ≥80 years. The secondary aim was to determine the appropriate surgical treatment of breast cancer in patients aged ≥80 years.

**Methods:**

We reviewed the medical records of 154 patients aged ≥80 years with early-stage breast cancer (mean age 83 years) who underwent surgery at the tertiary cancer center in the period from 2000 to 2008. Tumor stage was pT1/pT2 and pT3/pT4 in 75% and 25%, respectively. Surgical treatment comprised: quadrantectomy (in 27%), mastectomy (in 73%), axillary dissection (in 57%), and sentinel lymph node biopsy (in 18%), while 25% of patients had no axillary surgery.

**Results:**

During a median follow-up of 5.3 years, 31% of patients died of breast cancer, while 28% of patients died of other causes. Half of our patients with poorly differentiated breast cancer or estrogen receptor-negative tumor died of breast cancer. Multivariate statistical analysis showed that the pathological T-stage, pathological N-stage and estrogen receptors were independent prognostic factors for the duration of breast cancer-specific survival of patients.

**Conclusion:**

Short breast cancer-specific survival indicates that, in patients aged ≥80 years, breast cancer with metastases in axillary lymph nodes can be an aggressive disease.

## Background

The population of elderly people is increasing [1], and so is the number of elderly breast cancer patients. According to the data of the Slovenian Cancer Registry for 2008, 11% of all breast cancer patients were aged more than 80 years [2]. According to the data of the Statistical Office of the Republic of Slovenia, life expectancy in 2010 was 9.02 years for women aged between 80 and 84 years and 6.26 years for women aged 85 years or older [3]. Unfortunately, there is no consensus or guidelines on how to treat elderly breast cancer patients [4].

The aim of our retrospective study was to identify independent prognostic factors for the duration of breast cancer-specific survival of surgically treated patients aged ≥80 years. The secondary aim was to determine the appropriate surgical treatment of breast cancer in patients aged ≥80 years.

## Methods

This study included 154 patients who underwent surgery in the period from 2000 to 2008, when they were aged 80 years or older. Their medical records were reviewed. Data on the extent of the disease, pathomorphology of the tumor, treatment method, extent of breast and axillary lymph node surgery, complications after the surgery, disease recurrence, cause of death, length of survival, and length of breast cancer-specific survival were collected. The cause of death was determined from the data collected from death certificates. Postoperative complications were those observed within three months of the surgical procedure.

In the period from 2000 to 2008, a total of 469 patients aged 80 years or older were treated at the Institute of Oncology Ljubljana (IOL). As many as 437 (93%) of these received their first treatment at the IOL. Patients were not randomly selected for surgical therapy. In the majority of cases, a surgical procedure was proposed but declined by the patients or their relatives, therefore the majority of them were treated by hormonal therapy only. Altogether, 403 patients had locally or locoregionally limited disease. Before treatment, distant metastases were found in 34 patients.

Disease stage was determined according to the 7th edition of the TNM classification from 2010 [5]. Stage of the disease was unknown in 38 patients who did not have lymph node surgery and were clinically without suspicious or metastatic lymph nodes. Prior to the surgical procedure, all patients underwent chest X-ray. Skeletal scintigraphy was performed in 60 patients, and 21 patients had an ultrasound examination of the abdomen.

Considering their physical condition, the patients were grouped into four categories according to the classification of the American Society of Anesthesiologists [6]. Patients were further divided according to whether or not they received surgical treatment in line with the guidelines established at the IOL [7]. With regard to lymph node surgery, the patients were categorized into three groups: no lymph node surgery, sentinel lymph node biopsy only, and lymphadenectomy. When more than the sentinel lymph node was required, the standard practice was formal levels 1 and 2 axillary lymph node dissection. In patients with evident metastases in level 3 of the axilla, all three levels of lymph nodes were dissected.

Generally, the guidelines for breast cancer therapy established at the IOL followed the current consensus statements of the St. Gallen and the European Society of Surgical Oncology, the European Society of Medical Oncology, as well as the European Society of Radiotherapy and Oncology. Of course, these guidelines were regularly updated during treatment of our patients. According to the institutional guidelines for postoperative radiotherapy, all patients aged 70 years or less underwent whole-breast external beam radiotherapy in case of breast conserving surgery, with at least a 2-mm tumor-free surgical margin. Furthermore, all patients with a tumor larger than 5 cm after mastectomy, tumors without a clear surgical margin and/or with more than 3 metastatic lymph nodes had external beam radiotherapy of the thoracic region and regional lymph nodes. However, according to the institutional guidelines, radiotherapy could be avoided in patients older than 70 years in case of a large surgical margin, a tumor smaller than 2 cm, and in case of a low or moderate tumor grade which was hormone-dependent. However, in patients aged ≥80 years, postoperative radiation therapy was often omitted also in other circumstances, especially if the patient was not willing to undergo radiotherapy.

Our study was reviewed and approved by the Institutional Review Board of the Institute of Oncology Ljubljana and was performed in accordance with the ethical standards laid down in an appropriate version of the 1964 Declaration of Helsinki. Our study was conducted with the understanding and consent of the subjects. During the first admission to our Institute or during a follow-up visit, all of our patients are asked to give consent for the use of their chart and/or bioptic material for scientific purposes. Since the Institutional Review Board of the Institute of Oncology Ljubljana approved this specific study, our patients were not asked to give written consent for this specific study.

Univariate analysis was used to identify factors associated with disease-free and disease-specific survival. Disease-specific survival and disease-free interval were compared by a log-rank test. All comparisons were two-sided, and a p-value of <0.05 was considered statistically significant. Survival curves were calculated according to the Kaplan–Meier method. Cox’s multivariate regression model was used to identify independent prognostic factors of disease-free and disease-specific survival. The univariate and multivariate statistical analyses of the length of survival in breast cancer patients were performed using the SPSS 16.0 software for Windows (SPSS; Chicago, IL).

## Results

The data on patients and treatment methods are presented in Table 1. The patients were aged 80–90 years (mean age 83 years). Breast carcinoma was detected by clinical examination and imaging in 82% and 18%, respectively. There was no difference between the means of detection in older versus younger age groups (p = 0.54).

**Table 1:** **Characteristics of patients and treatment and univariate analysis of breast cancer specific survival**

Characteristic	Subgroup	Not dead due to breast cancer	Dead due to breast cancer	Univariate analysis
		(N = 106)	(N = 48)	
**American Society of Anaesthesiology score**	1 or 2	71	29	0.43
	3 or 4	35	19	
**Eastern Cooperative Oncology Group performance status**	1 or 2	94	40	0.36
	3 or 4	12	8	
**Preoperative hormonal treatment**	Yes	11	9	0.15
	No	95	39	
**pT-stage**	pT1 or pT2	86	29	0.006
	pT3 or pT4	20	19	
**pN-stage**	pN0 or unknown	69	14	0.001
	pN1 or pN2 or pN3	37	34	
**Breast cancer surgical procedure**	Quadrantectomy	32	10	0.23
	Mastectomy	74	38	
**Axillary lymphadenectomy**	Yes	51	36	0.002
	No	55	12	
**Lymph node surgery**	Without	31	7	0.003
	Sentinel node biopsy	24	4	
	Lymphadenectomy	51	37	
**Tumor differentiation**	1 or 2	71	19	0.003
	3	35	29	
**Oestrogen receptors**	Negative	9	11	0.014
	Positive	97	37	
**Progesterone receptors**	Negative	29	35	0.97
	Positive	77	13	
**Molecular subtype**	Hormone positive	96	38	0.047
	Triple negative	5	8	
	HER-2 positive	5	2	
**Surgery in accordance with guidelines**	Yes	47	8	0.001
	No	59	40	
**Adjuvant systemic therapy**	Tamoxifen	44	9	-
	Aromatase inhibitor	49	33	
	Cytostatics	0	1	
	Trastuzumab	0	0	
	Without	13	5	
**Radiotherapy**	Yes	10	7	0.34
	No	96	41	

Neoadjuvant hormonal therapy was used in 13% of patients. Breast-conserving surgery, mastectomy and postoperative radiotherapy were done in 27%, 73% and 12% of patients, respectively. Axillary lymphadenectomy, sentinel node biopsy and no axillary surgery were performed in 57%, 18% and 25%, respectively. In our patients, the following deviation from the guidelines for surgical treatment occurred in 53 cases: omission of the sentinel lymph node procedure in 38 patients, omission of lymphadenectomy in 11 patients with metastatic lymph nodes (5 with clinically evident metastatic lymph nodes and 6 with positive sentinel lymph nodes), and omission of reoperation because of positive or close surgical margins in 4 patients.

By means of pathomorphological examination, we were able to determine that the cancer measured 5–150 mm in diameter (arithmetic mean 37 mm, median 25 mm). Tumor stage was pT1/pT2 and pT3/pT4 in 75% and 25%, respectively. pN1/pN2/pN3 and pN0/unknown were reported in 54% and 46% of patients. Among 71 patents with positive lymph nodes, pN2 and pN3 were present in 18 and 13 cases, respectively. Stage I, stage II, stage III/IV, and unknown stage of breast carcinoma were present in 17%, 36%, 35% and 12% of patients, respectively. With regard to molecular subtype, the tumor was hormone-positive, triple-negative and HER-2 positive in 87%, 8% and 5%, respectively. There were no differences in surgical therapy between the molecular subtypes of breast cancer.

After the surgical procedure, the patients were followed up from 0.1 to 13.1 years (median 5.3 years). During this period, breast cancer recurred in 25% of patients. Five patients had local, regional and distant recurrence, twelve distant and local recurrence, four patients developed distant and regional recurrence, sixteen patients only distant, while two patients had only local recurrence. Five-year breast cancer-specific survival was 83%. A total of 31% of our patients died of breast cancer, while 28% of patients died of other causes.

Postoperative complications were observed in 25 (16%) of patients. Five (3%) of them suffered serious, life-threatening complications. On the first day after the surgical procedure, one patient experienced a myocardial infarction, which led to her death. Two patients suffered a cerebrovascular insult, and one of them also developed pulmonary embolism. During the surgical procedure, the patient with the locoregionally advanced cancer developed an iatrogenic pneumothorax. On the first day after the procedure, one patient was found to be bleeding into the wound and had to undergo another surgical procedure. Five patients were re-hospitalized for late complications: four of them had a wound infection and one came with an obstructed drain tube.

**Figure 1:**
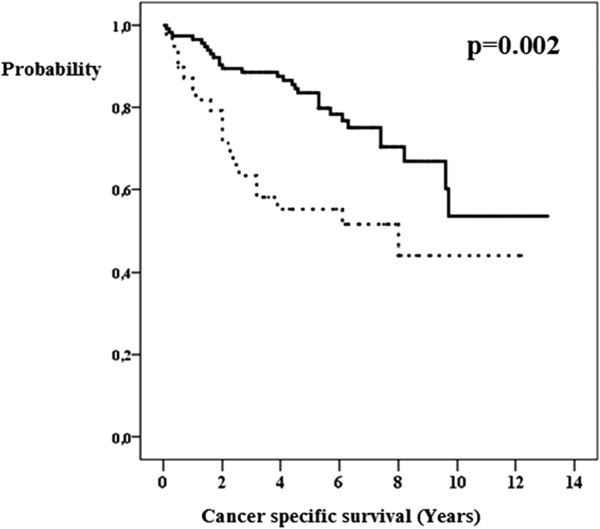
**pT-stage and breast cancer specific survival.** pT1 or pT2 (bold line). pT3 or pT4 (dashed line).

**Figure 2:**
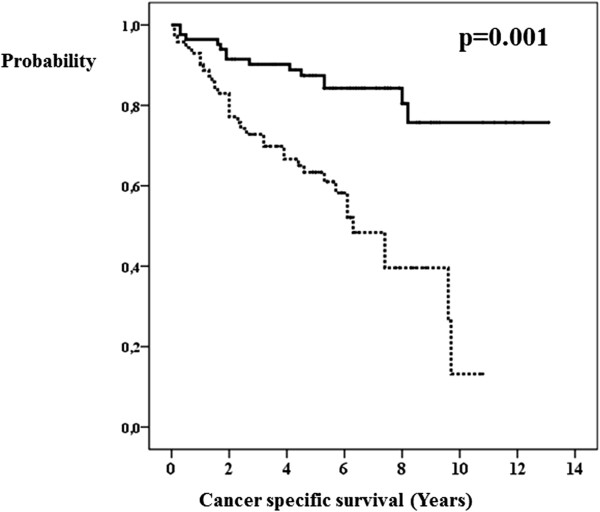
**pN-stage and breast cancer specific survival.** pN0 or not known (bold line). pN1 or pN2 or pN3 (dashed line).

**Figure 3:**
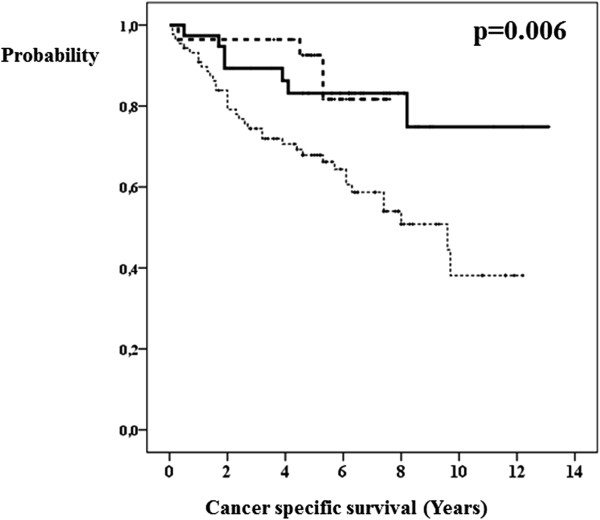
**Axillary lymph node surgical procedure and breast cancer specific survival.** Without lymph node surgery (bold line). Sentinel node biopsy only (dashed line). Lymphadenectomy (dotted line).

**Figure 4:**
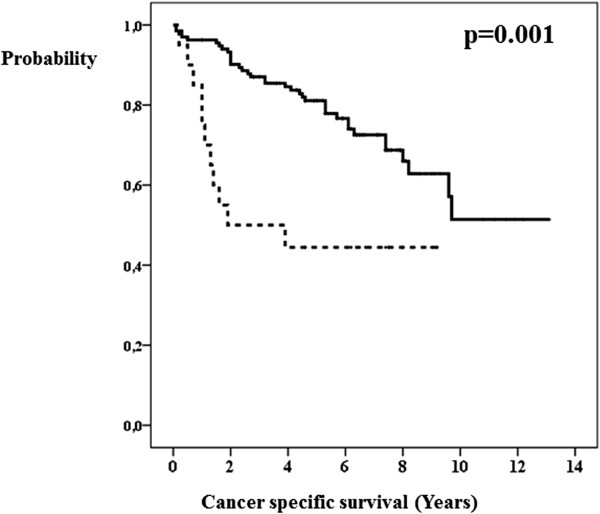
**Estrogen receptors and breast cancer specific survival.** Estrogen receptor positive tumor (bold line). Estrogen receptor negative tumor (dashed line).

The univariate analysis showed that the length of survival of breast cancer patients correlated with the following factors: pathological T-stage (Figure 1), pathological N-stage (Figure 2), axillary lymphadenectomy, lymph node surgery (Figure 3), estrogen receptors (Figure 4), degree of tumor differentiation, molecular subtype, and surgical treatment according to the established guidelines (Table 1).

All of the above-mentioned factors were included in the multivariate analysis (Table 2). Using the multivariate statistical analysis, we found that the pathological T-stage, pathological N-stage and estrogen receptors are independent prognostic factors for the duration of breast cancer-specific survival of patients. Patients with T3 or T4 tumors have a 1.04-times higher risk of shorter survival due to cancer compared to patients with T1 or T2 tumors. Patients with regional metastases have a 4.6-times higher risk of shorter survival due to cancer compared to patients with no metastases. Patients without estrogen receptors in the tumor have a 3.9-times higher risk of shorter survival due to cancer compared to patients with estrogen receptors in the tumor.

**Table 2:** **Results of multivariate analysis of breast cancer specific survival and independent factors for length of survival (p < 0.0001; 3 degrees of freeedom; -2 log likelihood = 396.46; chi-square = 44.78)**

Characteristics	Subgroup	Relative Risk	Confidence Interval 95%for Relative Risk
**pT-stage**	pT1 or pT2	1	
	pT3 or pT4	1.045	1.017 – 1.074
**pN-stage**	pN0 or unknown	1	
	pN1 or pN2 or pN3	4.589	2.393 – 8.799
**Estrogen receptors**	Positive	1	
	Negative	3.935	1.940 – 7.984

## Discussion

In our patients, the mean and median tumor size was 37 mm and 25 mm, respectively. Vetter et al. reported that the patients aged 80 years or older had larger median tumor size at diagnosis (25 mm vs. 18 mm) and higher disease stages compared to younger patients [8]. In our patients, breast carcinoma was detected by clinical examination and imaging in 82% and 18%, respectively. This is comparable to the results reported in the literature. The tumors of older patients were more often detected by clinical examination (39% vs. 17%) and less often by mammography/sonography (10% vs. 30%) [8]. However, screening mammography in patients aged 80 years or more is controversial [9–11].

There are no specific recommendations in the literature concerning the extent of surgical procedure performed to the breast and axillary lymph nodes in breast cancer patients aged 80 years or older [12]. The question that arises is whether to remove the entire breast and what to do with the axillary lymph nodes where no metastases were detected [12]. Multivariate analysis of the data on surgical treatment of our 154 patients showed that the pathological T-stage, pathological N-stage and estrogen receptors in the tumor were independent factors associated with the duration of breast cancer-specific survival of patients. The multivariate analysis also included data on the extent of breast and axillary lymph node surgery and data on the implementation of surgical treatment in line with the established guidelines. However, none of these factors were independent, which favors the decision of surgeons to adjust the extent of surgical treatment to the stage of the disease and the general condition of the patient. At the IOL, mastectomy was performed in 73% of cases. In the USA, mastectomy was performed in less than 40% of patients aged 80 years or older with stage I disease and in approximately 62% of patients with stage II disease [13]. In the US, following breast-conserving surgery in stage I and II of the disease, breast irradiation was performed in 31% of patients with stage I cancer and in 15% of patients with stage II cancer [13]. Due to a high proportion of patients who underwent mastectomy, irradiation was performed only in 12% of our patients.

There is a confounding variable that the increased use of axillary surgery is likely a surrogate for more advance disease. Most studies to date, however, have shown that regional disease in the axilla portends a worse prognosis, upon which surgical management of the axilla has no impact. Prognosis of patients is determined by standard of adjuvant care medical therapy. Considering lymph node surgery, our data are similar to those from the USA and the Netherlands. Lymph node surgery was performed in the Netherlands, at the MD Anderson Cancer Center, and at the IOL in 71% [14], 71% [12] and 75% of breast cancer patients, respectively.

In elderly patients, the treatment method must be selected also based on their life expectancy and concomitant diseases threatening their health. Safety of the surgical procedure or anesthesia can be assessed by the surgeon or anesthesiologist using the physical status classification of the American Society of Anesthesiologists (ASA) [6]. In patients with the ASA physical status 4, anesthesia is a very dangerous procedure, whereas it is safe in patients with ASA physical status 1 or 2. After the surgical procedure, five (3%) of our very old patients experienced serious and life-threatening health complications. Three of these patients were categorized into the ASA 2 group, and two of these three did not have a history of high blood pressure or cardiovascular disease. However, one of them developed a cerebrovascular insult and pulmonary embolism, while the other suffered a myocardial infarction on the first day after the surgery and died of it later on. At the MD Anderson Cancer Center, complications after the surgical treatment were reported in 6% (11/188) of their patients aged more than 80 years, and one patient died after the surgical procedure [15]. In order to better assess the risk associated with anesthesia or surgery and the patient’s life expectancy, the decision on the type of treatment should be based on a geriatric assessment which includes data on the functional, nutritional, cognitive and psychological status of the patient as well as her social status and social activities, including the information on comorbidities and concomitant medications [16].

Many patients aged 80 years or older die because of breast cancer. This was confirmed also by the present study: 31% of patients died of breast cancer during the median follow-up period of 5.3 years. In the United Kingdom, mortality due to breast cancer in women aged 80 years or older was 39% in the period from 1999 to 2009 [17]. Data on the survival of patients in the USA show that patients with early-stage breast cancer aged 80 years or older are at a higher risk of dying due to breast cancer than younger patients [13]. The higher morbidity is attributed to the fact that, compared to younger patients, elderly patients are rarely treated with cytostatics or receive less effective treatment schedules with fewer adverse events. However, the characteristics of tumors in elderly patients are similar to those in post-menopausal patients younger than 70 years [13]. Our results are consistent with this finding, as half of patients with poorly differentiated breast cancer or estrogen receptor-negative tumor died of breast cancer. Therefore, elderly patients with an aggressive tumor and/or locoregionally advanced breast cancer should probably also be treated with cytostatics. Yet, only one percent of our patients were treated with cytostatics. In the USA, among patients with stage I or stage II breast cancer with a hormone-negative tumor and positive lymph nodes, treatment with cytostatics was administered to 38% of patients aged 80–84 years and 10% of patients aged 85 years or older [13].

Hormonal treatment was preferred over surgery at the IOL in the period from 2001 to 2004 [18]. A total of 61% of 221 patients with early-stage breast cancer underwent hormonal treatment alone [18]. By means of multivariate analysis, we found that surgical treatment was an independent prognostic factor for longer survival, increasing the relative possibility of longer survival by 2.1 times [18]. The median overall survival was 83 months for patients treated with surgery, 57 months for patients who underwent surgery after neoadjuvant hormonal treatment, and only 33 months for those who had no surgery [18]. However, our study was not randomized; therefore its findings should be assessed accordingly [18]. It is possible that patients with more advanced disease were not treated surgically [18]. Recommendations for hormonal treatment only were based on the results of a randomized clinical trial EORTC 10851 comparing tamoxifen alone with modified radical mastectomy in patients aged 70 years or older [19]. The EORTC 10851 showed that hormonal treatment results in faster disease progression compared to surgical treatment. However, there was no difference in the overall survival between the two treatment groups in terms of breast cancer [19]. Following the findings of the EORTC 10851 study, breast surgery was performed only in 38% patients with locally or regionally limited cancer who received their first treatment at the IOL. Surgical treatment was performed considerably less often than in other studies. According to the data of the Dutch Cancer Registry, as many as 83% of patients aged 80 years or older with stage I or stage II breast cancer underwent surgery in the Netherlands in the period of 2001–2006 [14]. In the USA , surgery was performed in more than 98% of patients with stage I or stage II disease between 1992 and 2003 [13].

A total of 36% of our patients were not treated in line with the guidelines for the treatment of patients with breast cancer. Van Leeuwen et al. found that patients who underwent partial breast-conserving surgery without radiation therapy had a higher rate of locoregional recurrence than patients who were treated with surgery plus radiation therapy [12]. They also observed longer survival in breast cancer patients who underwent axillary lymphadenectomy as compared to those who did not undergo lymphadenectomy [12]. Contrary to their findings, our results show that there was a higher mortality among patients who received surgical treatment in line with the guidelines and those who underwent lymphadenectomy than among patients not receiving surgical treatment in line with the guidelines and those without lymphadenectomy. Surgeons at the IOL therefore utilized a more radical approach in patients with more advanced and more aggressive cancer. This surgical approach is in agreement with the modern concept of tailored treatment for breast cancer patients [20].

## Conclusions

Relatively long life expectancy of breast cancer patients aged 80 years or older presents us with new challenges. Using multivariate statistical analysis, we found that the pathological T-stage, pathological N-stage and estrogen receptors are independent prognostic factors for the duration of breast cancer-specific survival of patients. In this study, we found that our surgeons appropriately adjusted the extent of treatment according to the aggressiveness and extent of cancer and the biological age of the patient.
